# Identifying the needs for competency-based education in Europe: a needs assessment of cardiologists across 52 countries

**DOI:** 10.1080/21614083.2017.1337478

**Published:** 2017-06-22

**Authors:** S. Murray, C. Carrera, P. Lazure, P. Vardas, J. L. Zamorano, P. Kearney, L. Goncalves, K. Fox, A. Vahanian

**Affiliations:** ^a^ AXDEV Group Inc., Brossard, Canada;; ^b^ Education Department, European Society of Cardiology, Sophia Antipolis, France; ^c^ Performance Improvement Research Division, AXDEV Group Inc., Brossard, Canada; ^d^ Cardiology Department, Heraklion University Hospital, Heraklion (Crete), Greece; ^e^ Cardiology Department, University Hospital Ramon y Cajal, Madrid, Spain; ^f^ Cardiovascular Division, Cork University Hospital, Cork, Ireland; ^g^ Cardiology Department, University Hospitals of Coimbra, Coimbra, Portugal; ^h^ Cardiovascular Medicine, Imperial College, London, UK; ^i^ Cardiology Department, Bichat-Claude Bernard Hospital, Paris, France

**Keywords:** European Society of Cardiology, needs assessment, cardiology, collaboration, communication, skills, education

## Abstract

**Objective**: This international needs assessment was mandated by the European Society of Cardiology (ESC) to obtain an in-depth understanding of the current gaps and challenges of European cardiology professionals, with the aim to provide evidence for the development of needs-driven educational and professional development activities.

**Methods**: This ethics-approved needs assessment was conducted among cardiologists from all sub-specialties across 56 countries of Europe and the Mediterranean basin. A mixed-methods research approach was used, combining qualitative in-depth interviews and focus groups with a quantitative survey.

**Results**: Seventy-four (74) cardiologists participated in the qualitative phase and 866 completed the survey. Respondents represented 52 of the 56 targeted countries. Three themes were identified: 1) Challenges in the clinical decision-making process, 2) Challenges in establishing the patient-physician relationship, and 3) Sub-optimal team communication and collaboration. Specific gaps and causalities related to each challenge were found. Although most of the gaps were common across countries and sub-specialties, some significant differences were noted.

**Conclusion**: The findings of this needs assessment indicate gaps and challenges in clinical practice across countries and across sub-specialities. Taking cardiology as an example, this study identifies clear areas of focus, especially around issues of collaboration and communication, for targeted competency-based education in Europe.

## Background

The ageing population in Europe and the increased prevalence of chronic conditions such as obesity, diabetes, cancer and heart disease have made the provision of optimal care to patients more complex than ever across many specialities of medicine [1–3]. The practice of cardiovascular medicine is no exception. As the epidemiology of cardiovascular disease continues to change, today’s cardiologists are increasingly caring for patients with multiple chronic conditions and comorbidities. This situation is driving the need for effective interdisciplinary healthcare teams [4,5]. Evidence indicates that interdisciplinary teams meet the needs of patients more effectively, and do so in a more cost-effective and timely manner [6]. However, the associated competencies for team-based practices, including the domains of interpersonal and communication skills and collaborative practice outlined by Wood et al. [7], are not yet optimal among healthcare providers, hindering the full implementation of the interprofessional care model.

In response to this shift in the healthcare environment, the field of medical education had to evolve to gradually incorporate more competency-based education, through educational activities focused on communication skills and interprofessional practice [8–10]. However, the extent to which competency-based education is incorporated in the educational offerings, and translates to clinical practice in various medical specialities, varies greatly internationally [11].

A study was designed to better understand the current needs, challenges and successes of cardiology professionals in Europe, using the large network of professional members of the European Society of Cardiology (ESC), which represents over 95,000 healthcare providers specialised in cardiovascular diseases across 56 countries of Europe and the Mediterranean basin.

Taking cardiology as an example, the aim of this needs assessment was to assess the challenges faced by medical professionals and to highlight how speciality societies and other educational providers can gather evidence to better support healthcare professionals with needs-driven continuous learning opportunities.

## Methods

### Overview

This needs assessment study was included as part of a broader assessment that also investigated the organisational needs of the ESC, and utilised a mixed-methods approach combining qualitative and quantitative data in order to obtain an in-depth understanding of the perception of participants [12]. Participants also included leaders from national cardiac societies, representatives of the industry, and key influencers/decision-makers in the cardiology field. However, for the purpose of this article, which focuses on the educational needs and challenges of cardiologists, only the data collected amongst the cardiologist sub-group will be presented.

Interviews with open-ended questions and focus groups (qualitative phase) were conducted with general and sub-specialised cardiologists from a sub-set of nine targeted countries. Themes that emerged from the analysis of the qualitative data drove the design of an online survey (quantitative phase) that was deployed across 56 countries in Europe and the Mediterranean basin.

The study design, protocol and tools were developed by an independent research organisation specialised in behavioural and performance research (AXDEV Group, including co-authors SM and PL). Clinical expertise and contextualisation of the data were provided by subject matter experts comprised of cardiologists and decision-makers of the ESC (including co-authors PV, JLZ, PK, LG, KF and AV). Interpretation of the final data was provided by all co-authors.

### Recruitment and inclusion criteria

Cardiologists in the qualitative phase (interviews and focus groups) were recruited among the membership of ESC national cardiac societies in nine countries. The first five countries selected were the countries with the largest memberships within the ESC national cardiac societies (France, Germany, the UK, Spain and Italy). In addition, to ensure a representation of all regions of Europe and the Mediterranean basin in the qualitative phase, Poland, Russia, Sweden and Egypt were also included. To be eligible, potential respondents had to have a minimum of 5 years of cardiology practice experience and to be currently actively practicing (not only involved in research or holding an administrative position). For the quantitative phase (survey), cardiologists were recruited from the 56 member countries of the ESC.

Purposive sampling was used in both phases to ensure a representation of the different sub-specialities of cardiologists, to ensure a wide variety of practice settings (academic versus community, and rural versus urban) and years of clinical experience. All sub-specialities of cardiology were included in the study.

Participants were recruited using lists provided by the ESC. Invitations were sent by email with a link to an informed consent and a screening questionnaire to determine eligibility to participate in either the qualitative or the quantitative phase.

### Ethics

Study protocol approval was obtained from an independent ethical review board (IRB). Informed consent was completed by each participant prior to their participation. Participants to the qualitative phase received a financial compensation for their time. Participants to the online survey were entered in a draw, for a chance to win one of 13 one-year subscriptions to one of the ESC’s journals.

### Data collection and analysis

In the qualitative phase, participants were invited to participate in either a 45-minute semi-structured interview or a 90-minute virtual focus group. Each virtual focus group was formed of 2–4 cardiologists, practising in different countries but with the same type of practice setting (academic vs. community). Interviews and focus groups were conducted by trained facilitators. Broad open-ended questions on different domains were asked; in particular, different aspects of clinical practice (diagnosis, treatment, management) were reviewed with participants to identify potential challenges and causalities for these challenges. Qualitative data were collected in French or English.

After each interview or focus group, the trained facilitator filled in a data sheet with information on the relevance of the data and emerging themes. These data sheets were received by the co-author responsible for the first analysis (PL). Debrief sessions with facilitators were also conducted by PL and SM after the qualitative data collection phase to discuss the emerging themes and develop the coding tree for the in-depth qualitative analysis. Qualitative data of the interviews and the focus groups were coded using N-Vivo 7.0 software (QSR International, Cambridge, MA). The qualitative analysis process used consisted in a four-step approach integrating both thematic and directed content analysis [13,14]. The four steps were: (1) identifying codes within each of the four key domains based on the interviewer’s debriefing; (2) coding transcripts according to the developed coding structure; (3) developing new codes for data that did not fit the predefined codes; (4) identifying key emerging themes from the data. These themes were used to inform the design of the quantitative survey.

The quantitative data collection phase consisted of a 20-minute online survey. The survey questions allowed for validation of the challenges and needs that emerged from the qualitative data. Participants were asked to report on their levels of (1) knowledge, (2) skills and (3) confidence regarding clinical practice issues. Participants were also asked about (4) their clinical behaviours and (5) perceptions of certain topics, using ranking and agreement scales. The survey used branching questions based on each participant’s sub-speciality, as some questions were specific to one or more of the sub-groups of cardiologists.

The quantitative data of the online survey were analysed with SPSS 22.0 software (IBM Corporation, Armonk, NY) using frequencies, cross-tabulations and means. To investigate sub-group differences, a Pearson chi-square was calculated regarding the years of practice (≤20 years vs. 21+ years) and clinical setting (academic- vs. community-based). To assess for regional differences, grouping of countries into larger regions was done to obtain sufficiently large sub-samples to perform group comparison. Fifty countries were grouped into 18 regions. Grouping was done on the basis of geography proximity, culture and health systems. Four countries (France, Poland, Turkey and Ukraine) were kept as single-country regions, because of lack of similarity with their geographical neighbours based on the aforementioned criteria (culture, health system), and expected sample size being sufficient to be part of the group analyses. Six countries (Cyprus, Iceland, Israel, Lebanon, Malta and Syria) were excluded from these groupings, due to lack of sufficient shared characteristics with neighbouring countries and small expected samples. Responses from these participants were included in all other analysis.

The specialities of cardiologists were grouped into eight clinical areas of investigation. These clinical areas were developed by an oversight committee of cardiology experts, which included co-authors PV, JLZ, PK, LG, KF and AV. All sub-specialities which did not fall into one of the eight clinical areas were grouped into the “other” category, and general cardiologists were kept as their own separate category. The “other” category was too heterogeneous to be used in the sub-group analysis, but participants in that category were included in the full analysis. Table 1 presents the grouping of sub-speciality per areas of investigations. Analysis of variance (ANOVA) was applied to detect differences between regions and sub-specialities. Threshold for significance was set at α = 0.05.

**Table 1.** Description of the study sample (*n* = 940).

Sub-speciality	*n*
General cardiology/no sub-speciality	271
Arrhythmias	119
Cardiac imaging/e-technology	126
Heart failure/left ventricular dysfunction	78
Hypertension/pharmacology and pharmacotherapy	34
Interventions/peripheral circulation/stroke/surgery	85
Ischaemia/coronary artery disease/acute cardiac care/acute coronary syndromes	119
Prevention/rehabilitation/sports/nursing/ageing	31
Valvular disease/pulmonary circulation/myocardial-pericardial/congenital heart disease and paediatric cardiology	44
Other	33
**Region**	**n**
Spain & Portugal	142
Germany, Switzerland & Austria	109
Italy/San Marino	96
Russia/Belarus	88
UK & Ireland	65
France	58
Poland	49
North Africa	49
Nordic Countries	53
Greece & Macedonia	37
Romania & Moldavia	35
Turkey	17
Bulgaria, Hungary, Czech Republic & Slovakia	29
Ukraine	17
Benelux	22
Slavic countries	28
Eurasian republics	26
Baltic republics	8
Other	12
**Work setting**	***n***
Community-based cardiologists	611
Academic-based cardiologists	329

To strengthen the validity of the findings, the qualitative and quantitative data were triangulated, a method consisting of combining different data sources or data types [12,15].

## Results

A total of 940 cardiologists and cardiology sub-specialists participated in the study. Qualitative interviews were conducted with 74 cardiologists and the survey was completed by 866 cardiologists. A higher proportion of survey participants were from a community setting rather than an academic setting. Table 1 presents the details of the study sample by regional grouping and by grouping sub-specialities. One of the 18 regional groupings had to be excluded due to a low sub-sample size (Baltic republics, *n* = 8). Four of the 56 countries were not represented in the final sample (Estonia, Iceland, Malta, Montenegro).

The integrated analyses of the qualitative and quantitative data and interpretation by the co-authors revealed three themes related to (1) challenges in the clinical decision-making process, (2) challenges in establishing the patient-physician relationship and (3) sub-optimal team communication and collaboration. Specific knowledge, skills and attitude gaps related to each of these challenges were found. They are described in this section, which presents selected data from the quantitative survey, supported by examples of qualitative interview excerpts.

### Challenges in the clinical decision-making process

Participants reported gaps in their knowledge and skills in relation to three aspects of the clinical decision-making process. First, they reported specific knowledge and skill gaps in relation to biomarkers and imaging techniques, as summarised in Table 2.

**Table 2.** Reported knowledge and skill gaps in the diagnosis of cardiac conditions.

	% Knowledge (not acceptable/could be improved)	% Skill (Needs significant/minor improvement)		
Sub-specialities	USE of biomarkers to guide decisions^a^	SELECT which biomarker to assess^b^	INTERPRET biomarker results^c^	INTERPRET imaging data^d^
General cardiology/no sub-speciality	35%	**57%**	**52%**	**74%**
Arrhythmias	**52%**	**65%**	**57%**	**65%**
Cardiac imaging/e-technology	41%	**65%**	**53%**	35%
Heart failure/left ventricular dysfunction	**50%**	**61%**	43%	**63%**
Hypertension/pharmacology and pharmacotherapy	44%	**54%**	**54%**	**75%**
Interventions/peripheral circulation/stroke/surgery	37%	38%	38%	39%
Ischaemia/coronary artery disease/acute cardiac care/acute coronary syndromes	27%	38%	30%	**55%**
Prevention/rehabilitation/sports/nursing/ageing	48%	**64%**	**66%**	**62%**
Valvular disease/pulmonary circulation/myocardial-pericardial/congenital heart disease and paediatric cardiology	40%	**52%**	**56%**	**57%**
Other	**55%**	**61%**	**57%**	**75%**
**Totals**	40%	**55%**	49%	**60%**
**Significant difference (ANOVA)**	***p* = 0.004**	**n.s.**	***p* = 0.001**	***p* < 0.001**

^a^Please select what best describes your knowledge of the use of biomarkers to guide therapeutic decisions.

^b^Please indicate your current level of ability/skill selecting which biomarkers need to be assessed for each patient.

^c^Please indicate your current level of ability/skill interpreting what the presence/absence of a biomarker means for diagnosis.

^d^Please indicate your current level of ability/skill accurately interpreting imaging data.

Forty per cent of participants reported sub-optimal knowledge of the use of biomarkers to guide their therapeutic decisions. This gap in knowledge was more frequently reported in certain sub-specialities than others (i.e. arrhythmia (52%) and heart failure specialists (50%)). The proportion of participants reporting their knowledge as either not acceptable or could be improved was also significantly higher in Eastern regions (e.g. Ukraine 59% and Bulgaria, Hungary, Czech Republic and Slovakia 59%) as compared to Western regions (e.g. France 28%; Germany, Switzerland and Austria 24%).

Second, half of the cardiologists (55%) also reported their skills to select the biomarkers or to interpret biomarker test results as needing minor or significant improvement. Variations across sub-specialities were observed, although they did not always reach significance (see Table 2 for details). No difference was observed between regions.

Sixty per cent of participants reported needing minor or significant improvement in their skills to accurately interpret imaging data. Significant differences were observed between sub-specialities, with higher gaps reported in general cardiology and “hypertension/pharmacology and pharmacotherapy”. Significant differences across regions were also observed (with a lower gap being found in the Bulgaria/Hungary/Czech Republic/Slovakia region, 43%, and highest gap in Ukraine, 88%).

Specifically, participants reported that making a diagnosis when having to integrate contradictory investigation results constitutes a challenge:

“*The challenge is when there is a discrepancy between data, clinical data and imaging data, (…) it is for us to distinguish between things. Is what we see on ultrasound or imaging correct or not? Is what you see really the status of the patient, or is it a coincidence?*”

- Academic Cardiologist, Cardiac Imaging, France

Third, participants reported being challenged in their decision-making process regarding the selection of treatment, due to lack of skills factoring in comorbidities, cost–benefit analysis, results of biomarker analysis and notion of quality of life. Table 3 illustrates the proportion of participants who reported a need for improvement in their skills for each of these factors per sub-specialities.

**Table 3.** Skill gaps integrating different factors in the decision making process.

Sub-specialities	% **SKILL** (Needs significant /minor improvement)			
	Comorbidities^e^	Cost^f^	Bio-markers^g^	Quality of life^h^
General cardiology/no sub-speciality	**56%**	**72%**	**55%**	**50%**
Arrhythmias	**57%**	**65%**	**70%**	**52%**
Cardiac imaging/e-technology	**52%**	**63%**	**61%**	**56%**
Heart failure/left ventricular dysfunction	**60%**	**68%**	**56%**	44%
Hypertension/pharmacology and pharmacotherapy	**50%**	**67%**	**57%**	**52%**
Interventions/peripheral circulation/stroke/surgery	**50%**	**63%**	49%	**53%**
Ischaemia/coronary artery disease/acute cardiac care/acute coronary syndromes	**52%**	**58%**	40%	**50%**
Prevention/rehabilitation/sports/nursing/ageing	43%	**78%**	**67%**	32%
Valvular disease/pulmonary circulation/myocardial-pericardial/congenital heart disease and paediatric cardiology	**54%**	**72%**	**67%**	**61%**
Other	**55%**	**74%**	**67%**	**66%**
**Totals**	**54%**	**67%**	**57%**	**51%**
**Significant differences between sub-specialities (ANOVA)**	n.s.	n.s.	***p* = 0.003**	n.s.

^e^Please indicate your current level of ability/skill considering all comorbidities (liver problems, diabetes, other) when recommending a treatment plan.

^f^Please indicate your current level of ability/skill considering the cost of each treatment option compared to its potential benefits.

^g^Please indicate your current level of ability/skill adapting treatment recommendations to biomarker analysis results.

^h^Please indicate your current level of ability/skill assessing treatment impact on patient’s quality of life to optimally inform treatment modifications.

The most frequently reported gap in skills was related to the assessment and integration of the cost-benefit analysis in the treatment decision-making process. Between 67% and 78% of cardiologists among every sub-speciality and region reported needing minor or significant improvement in this skill.

More than half (54%) of cardiologists reported needing minor or significant improvement in their skills to reach treatment decision-making with patients having comorbidities, as supported by this quote:

“*Often the patient has different diseases, and diseases have to be treated together, and that is often a challenging thing. Because if you want to treat that, you keep in mind that the patient has other diseases where sometimes you cannot treat it. And also the drug therapy is not easy in patients that have multiple diseases.*”

- Academic Cardiologist, Arrhythmia, Germany

Among the causalities for this gap, participants reported that clinical trial data are usually not available for patients with multiple comorbidities and/or on multiple medications, which hinders reliance on guidelines to guide their clinical decision-making process when treating these patients.

Cardiologists self-reported, to a lesser extent (51%), a lack of skills in assessing and integrating the patient quality of life in their treatment decision-making process.

### Challenges in establishing the patient-physician relationship

Participants reported challenges in ensuring an optimal patient–physician relationship. Specifically, sub-optimal skills regarding the communication with the patient were reported as evidenced by this participant:

“*Sometimes they can be wrongheaded. In that case, it can be difficult if you disagree with their choices, you want to explain, (…) if they still don’t want it, fair enough, but you have to make sure they understand it. Though on the other hand, there are patients who just go, yeah, whatever you like, whatever you think is best and don’t engage (…)I think it’s knowing how to deal with a spectrum of patients and it’s quite a skill.*”

- Community Cardiologist, Arrhythmia, UK

Indeed, the majority of cardiologists (59%) reported having sub-optimal skills to engage their patients to take a proactive role in the management of their disease. This gap was shared across sub-specialities. Although it did not reach significance, cardiologists in Western regions tend to report sub-optimal skills in engaging patients to take a proactive role more frequently than cardiologists in Eastern regions. For example, 48% of participants in Russia reported their skills needing improvement as compared to 83% in France.

Participants also reported sub-optimal skills in supporting their patients to make significant lifestyle changes due to their medical condition.

“*The first challenge is not to get too frustrated as a doctor because as we know, it’s much easier to get the patient to take some pills than really change his lifestyle. Second challenge is (…) trying together to change his lifestyle.*”

- Academic Cardiologist, Heart Failure, Germany

Over 60% of cardiologists reported their skills to support patients to implement lifestyle changes, need improvement. This skill gap was reported in similar proportions across sub-specialities but was present to a lesser extent in Eastern European countries than in Western countries.

Another reported gap in skills in relation to the patient-provider relationship concerns the ability to discuss with patients information they may have obtained from less credible sources, such as the internet, or family members, as this quote exemplifies:

“*There is information on the web, but it’s very general, but here I’m focusing on the things that the patients say and I’m trying to convince them that you must trust me, not the blog, or the tabloid, or your neighbour. So those are challenges.*”

- Community Cardiologist, General Cardiology, Sweden

Several contextual factors were reported by participants as adding to the challenge of communicating efficiently with patients. Among these contextual factors, the ageing of the population and low health literacy were reported to increase the difficulty to adequately transfer information to patients. Participants reported that adapting their communication in consideration of the patients’ characteristics is made even more challenging by the limited time they have for each consultation, an additional constraint imposed by systems thriving for cost-efficiency.

### Sub-optimal team communication and collaboration

Participants reported a lack of clarity of roles and responsibilities within the interdisciplinary team. Table 4 presents the level of agreement with three statements related to the definition of roles and responsibilities between different healthcare professionals in the co-management of cardiac patients. There was a low level of agreement with the statements related to clear definition of roles and responsibilities between all groups of healthcare professionals, and particularly between cardiologists and non-cardiologist specialists. In general, the definition of roles and responsibilities was reported to be clearer in the Eastern regions compared to Western or Central European countries.

**Table 4.** Clarity of roles and responsibilities and quality of collaboration.

Countries/region	France	Turkey	Benelux	Nordic Countries	Slavic countries	UK/IRE	North Africa	SPA/POR	ITA/SM	GER/SWI/ AUS	ROM/MOL	BUL/HUN/CZR/SLV	Poland	GRE/MAC	Ukraine	Eurasian republics
**% 4–5 Rating of clarity of roles and responsibilities in the co-management of patients by:**																
Different sub-speciality cardiologists* ^i^	52%	53%	50%	**47%**	50%	**42%**	66%	**44%**	**49%**	**44%**	62%	**45%**	61%	**42%**	**37%**	62%
Cardiologists & non-cardiology specialists* ^j^	50%	53%	**36%**	**46%**	50%	**41%**	**44%**	**25%**	**45%**	**31%**	62%	**21%**	**34%**	**33%**	**25%**	58%
Cardiologists and primary care* ^k^	**49%**	**43%**	**45%**	**34%**	**36%**	57%	51%	**22%**	**36%**	**34%**	57%	**48%**	**31%**	**41%**	**37%**	52%
**% 4–5 Rating of the quality of collaboration between specialists and GPs**																
Collaboration to jointly manage patients with a cardiac condition* ^l^	**27%**	**14%**	50%	**21%**	**15%**	**30%**	**17%**	**18%**	**38%**	**46%**	**29%**	**21%**	**5%**	**17%**	**6%**	**28%**
Quality and timeliness of referrals* ^m^	**25%**	**14%**	**32%**	**30%**	**19%**	**31%**	**14%**	**17%**	**18%**	**38%**	**31%**	**18%**	**3%**	**11%**	**6%**	**36%**

^i^The roles and responsibilities between the different sub-speciality cardiologists in the co-management of patients with multiple cardiac conditions are well defined (agreement scale where 1 = fully disagree to 5 = fully agree).

^j^The roles and responsibilities between cardiologists and non-cardiology specialists in the co-management of patients with comorbidities affecting multiple organs are well defined (agreement scale where 1 = fully disagree to 5 = fully agree).

^k^The roles and responsibilities between cardiologists and primary care in the co-management of patients with cardiac conditions are well defined (agreement scale where 1 = fully disagree to 5 = fully agree).

^l^Using the scale provided (where 1 = low, 5 = optimal and N/A = not applicable to my practice), please rate the quality of the collaboration between specialists and General Practitioners/Family Physicians to jointly manage patients.

^m^Using the scale provided (where 1 = low, 5 = optimal and N/A = not applicable to my practice), please rate the quality and timeliness of referrals from General Practitioners/Family Physicians.

UK/IRE: United Kingdom, Ireland; SPA/POR : Spain, Portugal; GER/SWI/AUS : Germany, Switzerland, Austria; ITA/SM: Italy, San Marino; ROM/MOL: Romania, Moldavia; BUL/HUN/CZR/SLV: Bulgaria, Hungary, Czech Republic, Slovakia; RUS/BEL: Russia, Belarus; Benelux: Belgium, Netherlands, Luxembourg; Nordic Countries: Sweden, Denmark, Finland, Norway; Slavic Countries: Serbia, Croatia, Bosnia and Herzegovina, Albania, Slovenia, Kosovo, Montenegro; North Africa: Morocco, Libya; Eurasian republics: Armenia, Georgia, Azerbaijan, Kyrgyzstan, Kazakhstan.

In absence of clear roles and responsibilities, participants also reported low quality and timeliness of the referrals they receive from other physicians. There was a significant difference across countries with Eastern regions rating quality higher, as compared to Western regions (see Table 4).

More than half (53%) of the survey respondents reported needing improvement in their skills to communicate within a multidisciplinary team in order to manage their patients efficiently. The following quote illustrates one of the factors that can impact interdisciplinary communication, namely the lack of common terminology between specialists and generalists:

“*We all speak different languages and in cardiology we are normally specialised from a high level, and if you talk to a generalist, you sometimes don’t realise you are talking about things your colleague doesn’t know or is not as clear for him.*”

- Community Cardiologist, General Cardiology, Germany

The clarity of the communication process between cardiologists and non-cardiologist specialists, as well as between cardiologists and primary care physicians was generally rated low (37% rated 4 or 5 on the five-point scale in both cases, data not shown).

“*I’d say we are not communicating well with different specialists, we have different interests, different focus … it’s more difficult to communicate with other different specialities.*”

- Community Cardiologist, General Cardiology, Poland

In general, the quality of the collaboration with other healthcare providers was also rated low by a majority of participants (see Table 4 for details). The overspecialisation of cardiology was mentioned by participants as a factor that could contribute to the poor quality of the collaboration and impact the communication processes within the field of cardiology.

“*Sometimes there are huge walls between the cardiologists in the same department, because it’s so specialised they are just seeing the things that they work with and they are not caring about other things.*”

- Community Cardiologist, General Cardiology, Sweden

## Discussion

Traditionally, medical training has focused on the acquisition of knowledge and skills that are intimately related to the disease state (or to treat symptoms). Increasingly, other sets of skills and competencies, such as interpersonal skills and professionalism, are being recognised as inherent parts of the education and training of medical professionals [16,17].

This study provides evidence of challenges experienced by cardiology specialists across 52 countries of Europe and the Mediterranean basin. It identifies clear areas of focus for targeted educational activities to facilitate cardiologists’ clinical decision-making processes on specific aspects of assessment, treatment and management of patients with cardiac conditions. It also highlights a need for cardiologists of all sub-specialities to bridge the gaps in relation to optimal patient–physician relationships and interprofessional collaboration and communication skills.

Selecting the appropriate biomarkers and interpreting imaging data are two examples of specific and specialised skills that are paramount to the provision of optimal care for any cardiology specialist and are required across all sub-specialities of cardiology, although to a different extent. Data from this study indicate that cardiologists recognise a necessity to improve their knowledge of biomarkers and may be interested in bettering their skills to properly select which biomarker test to perform and how to analyse the results to make the best treatment decision. Challenges in relation to integrating investigation results were more pronounced in certain specialities, which may indicate an opportunity to design tailored educational programmes to bridge those gaps for those sub-specialists.

Although the acquisition of the knowledge and skills inherent to the profession of cardiology is extremely important, the gaps reported by participants in this study were mainly related to specific skill sets. These skill sets are essential to develop a good relationship with patients and facilitate an optimal communication that would support patient engagement in their care and adherence to their treatment plan. The evidence of the presence of skill gaps over knowledge gaps supports the need for targeted education that uses interactive formats of learning, such as case-based learning, virtual patients or simulation [18,19].

Interdisciplinary collaboration is becoming more common in the medical practice to ensure an optimal management of complex patients with multiple comorbidities and chronic disease such as the elderly [4]. Cardiologists are now asked to collaborate with other cardiac specialities and with primary care physicians to manage multiple cardiac conditions or other types of comorbidities. Likely team behaviours can only be reinforced through opportunities to learn in a team setting. As early as 1998, it was identified that interprofessional education (IPE) was beneficial for healthcare providers and healthcare organisations as it was shown to enhance personal and professional confidence and promote mutual understanding between professions. It has also helped to reduce the occurrence of communication breakdowns and generally contribute to increase morale and efficiency of team members [20]. However, barriers to the implementation of IPE in Europe have been documented and include the lack of incentives towards these formats of learning, and a higher level of hierarchy remaining in the medical culture in many countries [21].

In a recent performance improvement initiative deployed in Spain in the field of diabetes, the presence of nurses alongside physicians in a training aiming to bridge the gaps in diabetes care in primary care clinics, was not well accepted by participants, who reported having preferred to be trained independently [22]. There is an opportunity to share with healthcare providers the evidence of IPE and the impact of adopting a multidisciplinary team approach to care, from an efficiency standpoint and from the patient perspective [6,20,23]. However, education alone would not be sufficient to address issues of sharing of roles and responsibilities or collaboration challenges between primary care and speciality care, which may be rooted in more complex health system issues.

In addition, the perceived relevance of certain sets of skills in relation to communication or collaboration (with patients and with other healthcare providers) may be influenced by the culture of medicine in each given region or country and the level of hierarchy in place in that local culture of medicine. For example, the findings of this study indicate lower skill gaps in relation to communicating with patients in Russia than in France, for example. However, other studies have indicated the importance of shared decision-making, for example, which could be considered as a proxy of patient-physician communication, and the factors that influence shared decision-making can vary across cultures [24,25]. Whilst the precise impact cultural differences may have had on the participants’ responses could not be determined in this study, it would be of interest for a future study to explore specifically how the perceived relevance of communication and collaboration skills varies from one region to another, and how this perceived relevance impact the perceived need for training. It appears logical, for example, that participants would report lower need for improvement for a skill, which they do not consider essential in the first place.

Finally, the findings indicate shared gaps and challenges across the different sub-specialities of cardiology and across countries, and also call for education that addresses topics such as the soft skills of communication, and focuses on other competencies that are as important nowadays as the core medical competencies. In North America, competency models in the health professions (such as the CANMeds model from the Royal College of Physicians and Surgeons in Canada or the American Board of Medical Specialists (ABMS) in the US) include interpersonal and communication skills [10,26]. In Europe, although the appearance of competency-based education is more recent, there has been an attempt to develop competency models that are specific to certain professions [8].

### Limitations

This study was conducted across 52 countries in Europe and the Mediterranean Basin and reached a large number of cardiologists to investigate their clinical challenges and educational needs. However, Europe is not a homogenous territory therefore regional findings must be interpreted with caution. Although the questions were designed by educational experts to limit the influence of cultural norms, the meaning of words may have been interpreted differently by participants for whom English is not their first language. Self-confidence is also a factor which varies greatly across culture, and could have influenced a respondent’s tendency to self-report a lack of knowledge or skills [27].

In most instances in this study, the percentage of participants who reported a need for improvement was over 50%, which indicates important opportunities to bridge gaps with proper educational activities. Multiple differences were observed across regions, although a clear pattern can only be hypothesised given the lower number of participants in some regions, limited the statistical power of the analyses. Our findings could be validated using larger sample size, if obtaining more precise information at a country level was judged necessary before deploying specific educational programmes.

### Conclusion

The findings from this study indicate shared gaps and challenges across the different sub-specialities of cardiology and across countries, especially around issues of collaboration and communication. This offers an opportunity for educators to develop evidence-based continuing education activities in Europe aiming at bridging those gaps, while continuing to also address specific knowledge and skills needs with proper educational formats. Those findings can also be used by cardiologists to reflect on their own practice gaps and needs that they would like to address, to improve the care they provide to their patients.
